# Bibliographical

**Published:** 1872-08

**Authors:** 


### BIBLIOGRAPHICAL.

LECTURES UPON DISEASES OF THE RECTUM, delivered in the Bellevue Hospital Medical College, session 1869–'75. By W. H. Van Buren, A. M., M. D., Professor of the Principles of Surgery, with Diseases of the Genito-Urinary Organs, etc., in the Bellevue Hospital Medical College, etc., etc. New York: D. Appleton & Co. 1872.

These lectures comprise the results of a number of years of observation by the learned author, both in hospital and private practice. He has succeeded in making a volume intelligible and practical. It will be found a valuable and convenient guide to the practitioner upon all diseases of the rectum.

HEALTH BY GOOD LIVING. By W. W. Hall, M. D., Editor of Hall's Journal of Health, etc., etc. Seventeenth Thousand. New York. Published by Hurd & Houghton, Cambridge: Riverside Press. 1872.

This volume is replete with directions for maintaining "good health," and valuable advice on "good living" as one means of warding off disease. The author says: "This book is to show how high health can be maintained and common diseases cured by 'good living,' which means eating with a relish the best food prepared in the best manner. The best food includes meats, fish, poultry, wild game, fruits, and the grains which make bread. The best cookery preserves the natural tastes and juices. As there can be no 'good living' without a good appetite, how to get this great blessing without money and without price necessarily, is pointed out, and it is hoped, in very clear and plain terms."

A HAND-BOOK OF UTERINE THERAPEUTICS, AND OF DISEASES OF WOMEN. By Edward John Tilt, M. D., Member of the Royal College of Physicians, Consulting Physician to the Farringdon General Dispensary, etc., etc. Second American Edition, thoroughly revised and amended. New York: D. Appleton & Co. 1869.

This valuable work is well known. The principal points developed in the work are: "Firstly, The paramount importance of hygiene for the relief and cure of diseases of women. Secondly, The constitutional nature of many diseases of women and the impossibility of curing them without constitutional remedies.—Thirdly, The manifest reaction of uterine diseases in the female system, and the impossibility of curing many uterine complaints without surgical measures. Fourthly, The great value of therapeutics to assuage and cure diseases of women, and the belief in the value of those remedial measures that are as old as medicine itself—such as venesection, emetics and caustics." The work should be in the hands of every physician.

PRACTICAL MIDWIFERY, and Obstetrics, Including Anæsthetics. By John Tanner, M. D., M. A., LL. D., Member of the Royal College of Physicians, etc., etc. Philadelphia: J. B. Lippincott & Co. 1871.

Everything from the pen of the lamented Tanner bears the impress of genius and learning, and the volume before us is no exception to the rule. As a hand-book it will be found of great value to students and practitioners.

THE CORRECT PRINCIPLES OF TREATMENT FOR ANGULAR CURVATURE OF THE SPINE. By Benjamin Lee, A. M., M. D. Philadelphia: J. B. Lippincott & Co. 1872.

This is a volume of seventy-five pages, and treats very fully and satisfactorily of curvatures of the spine. It is a practical little volume, and may be read with advantage by all practitioners.

EMERGENCIES AND HOW TO TREAT THEM. The Etiology, Pathology and Treatment of the Accidents, Diseases, and Cases of Poisoning, which Demand Prompt Action. Designed for Students and Practitioners of Medicine. By Joseph W. Howe, M. D., Visiting Surgeon to Charity Hospital, etc., etc. New York: D. Appleton & Co. 1871.

A work so useful and practical in character as the above should be in the library of every practitioner. The copious title fully explains its scope and design, as well as its practical value. It affords instructive reading, and abounds with information which all would do well to learn. We commend it to the profession.

THERMIC-FEVER, OR SUNSTROKE. By H. C. Wood, Jr., M. D., Professor of Medical Botany and Clinical Lecturer on Diseases of the Nervous System in the University of Pennsylvania, etc. Boylston Prize Essay. Philadelphia: J. B. Lippincott & Co. 1872. Through Messrs. Phillips & Crew, Atlanta, Ga.

This is an interesting volume of 128 pages. It is certainly the most valuable contribution to the medical literature of sun-stroke yet given the profession. The author divides his work into four parts, each part being elaborately and scientifically discussed. Part I. embraces the clinical history of sun-stroke; Part II., the nature of sun-stroke; Part III., its treatment; Part IV., the sequelæ of sun-stroke. The volume is worthy of the study of every scientific practitioner, furnishing much valuable information and abounding with practical thoughts.

*Selected Obstetrical and Gynæcological Works of Sir James Y. Simpson Bart, M. D., D. C. S.*, Late Professor of Midwifery in the University of Edinburgh; Containing the Substance of his Lectures on Midwifery. Edited by J. Watt Black, M. D., Member of the Royal College of Physicians, London, etc., etc. New York: D. Appleton & Co, 149 & 551 Broadway. 1871.

This volume contains "all the more important contributions of Prof. Simpson, to the study of obstetrics and diseases of women, with the exception of his clinical lectures on the latter subject." The work consists of six parts: Part I. embodies Lecture Notes, of great value; Part II, Pregnancy; Part III., The Fœtus and its Appendages; Part IV., Parturition; Part V., The Peurperal State; PartVI, Non-puerperal Diseases of Women. From the above, to those unacquainted with the scope of the work, its comprehensiveness and value may be drawn—but faintly so. The work should be seen to be fully appreciated—as all the works of the distinguished author must be, by the ardent student and scientific practitioner.

*The Physiology and Pathology of the Mind.* By Henry Maudsley, M. D., London, Physician to the West London Hospital, etc., etc. New York: D. Appleton & Co. 1872.

The aim of the author, as stated in the volume before us, was two-fold: first, to treat of mental phenomena from a physiological rather than from a metaphysical point of view; and secondly, to bring the manifold instructive instances presented by the unsound mind to bear upon th interpretation of the obscure problems of mental sciences." Ten years of practical study has given its valuable fruits in the present volume. The work is divided into two parts: The first part contains chapters "on the method of the Study of Mind; The Mind and the Nervous System; The Spinal Cord and Reflex Action; The Sensory Centres and Sensation; The Supreme Cerebral Centres and Ideation; On Emotion; On Volition; On Actuation; On Memory and Imagination."—Part Second contains chapters "On the Causes of Insanity; On the Insanity of Early Life; The Varieties of Insanity; The Pathology of Insanity; The Diagnosis of Insanity; The Prognosis of Insanity; The Treatment of Insanity."

This rapid and imperfect survey of the contents of the work will convey to the reader its great importance both as a volume for reference and of earnest study. It will be a valuable accession to every medical library.

*Obstetric Operations, Including the Treatment of Hemorrhage.* By Robort Barnes, M. D., London, F. R, C. P., Obstetric Physician to, and Lecturer on Midwifery and the Diseases of Women and Children at St. Thomas's Hospital, etc., etc. With Additions by Benj. F. Dawson, M. D., Late Lecturer on Uterine Pathology in the Medical Department of the University of New York, etc., etc. Second American Edition. New York: D. Appleton & Co. 1871.

The value of this work is such as should place it in the hands of every student and practitioner. In the language of the author—one of the most accomplished obstetricians and gynæcologists in this country—"the work will prove of the greatest value" to students and physicians, "being, as it is, a most perfect text-book on obstetric operations by one who has fairly earned the right to assume the position of a teacher." Cŏming from Dr. Dawson, no higher encomium could be given any work or teacher. The work fully merits the judgment thus passed upon it, and will be so esteemed by every practitioner who may read it.

*The Management of Infancy, Physiological and Moral.* Intended Chiefly for the Use of Parents. By Andrew Combe, M. D.—Revised and edited by Sir James Clark, Bart, K. C. B., M. D., F. R. S., Physician-in-Ordinary to the Queen. New York: D. Appleton & Co. 1871.

This little volume cannot fail to instruct all who may read it; and though specially designed for parents, it will nevertheless be highly appreciated by the profession at large—touching upon subjects, which as physicians, are constantly coming before them. We cordially commend it to all.

